# A Consideration of Actinomycosis

**Published:** 1892-05

**Authors:** F. S. Billings

**Affiliations:** Director of the Patho-Biological Laboratory of the State University of Nebraska


THE JOURNAL
OF
COMPARATIVE MEDICINE AND
VETERINARY ARCHIVES.

VOL. XIII. MAY, 1892. NO. 5.

### A CONSIDERATION OF ACTINOMYCOSIS AS TO ITS NATURE AND RELATION TO THE PUBLIC HEALTH.

BY DR. F. S. BILLINGS, Director of the Patho-Biological Laboratory of the State University of Nebraska.

As should be well known to every cattle breeder in the West, and every farmer who feeds cattle (and who of them does not in a more or less extensive manner), the Live Stock Commission of the State of Illinois began a senseless war upon the cattle breeding and feeding interests of the West by the wholesale condemnation of all cattle affected, not only with "lumps" on the jaws, but lumps of almost any kind, large or small, that are visible in the tissues around the jaws, on the general assumption that these "lumps" are caused by a micro-organism—a germ known to the medical profession as actinomyces. The ground of this wholesale condemnation was that actinomycosis is a "*dangerously contagious disease*," and the only reason the Illinois Live Stock Commission has for making such an assertion is, and has been, to use their own words, that "*the fungus, actinomyces, could be transmitted from one animal to another by inoculation, which fact establishes it as a contagious disease.*"

Another assertion, with which in fact and law this Commission has nothing to do, is that the fungus produces a condition of the flesh of the afflicted animals which renders it extremely dangerous as an article of human food, which makes the question at issue of equal importance to the medical profession as one touching intimately the public health.

Were lump jaw a "dangerous contagious disease," the Commission has certainly the power to act in the premises, but, as will be shown, it has contradicted that assertion by never acting or treating the disease as if it were "dangerously contagious," and, in fact, in the late trial at Peoria, its expert witnesses entirely gave up that point, hence, actually admitted that they were and had been going entirely beyond the authority given them by the law for the suppression of "dangerous contagious" diseases. As to the relation of the flesh of animals afflicted with "lump jaw" as an article of human food, that belongs to the Boards of Health and not the Live Stock Commission, and the sale of such meat is a matter for a criminal court to consider and not a civil one.

As is well known, the so-called "Whisky Trust," for whom the Illinois Live Stock Commission had condemned a very large number of cattle on account of "lump jaws," recently entered suit against that body in a civil court at Peoria, Ill., the writer being the one so-called "expert" to represent the Trust and the cattle breeding and feeding interests of the country, though others were also there in such capacity. The following letter from the attorneys of the Trust will give the public a suitable idea of the verdict rendered :

"PEORIA, Ill., Nov. 26, 1891.

"DR. F. S. BILLINGS, Lincoln, Nebraska

"DEAR SIR :—Our jury in the cattle case was discharged yesterday. It went out Monday evening, and remained out until yesterday forenoon, when they announced a failure to agree, and were discharged. This will necessitate another trial. When they went out they stood seven to four, seven for plaintiff. Day before yesterday three of the four agreed with the seven upon a verdict in favor of the plaintiff, the only difference being the amount of damage. One man, a little druggist, hung the jury. The effect of the trial seemed to be largely in our favor.

"Yours truly,

"STEVENS & HORTON."

*The Importance of this Case to the Cattle Breeders and Feeders as well as to the Consumers of the Country.*—There is not a single breeder or feeder of cattle in the Western States, or a consumer of meat, that does not belong to the wealthy classes, who should not have a direct and personal interest in the final results of this case which, though not with that intent, has been really taken up in their interest by the so-called "Whisky Trust." That this is in a measure the case, was fully demonstrated during the late trial by the numerous telegrams and letters received from farmers and breeders by both Mr. J. B. Greenhut of the Trust, and myself. The interest should be universal, however. It is not the limited number of cattle fed at the distilleries which are threatened. It is the entire cattle business of the great breeding and grazing States of the West, and the great army of consumers in the country whom everything tending to unnecessarily to affect the food supply most directly influences. It is not the "lump jawed" cattle of the great feeders any more than the one or two such of the ordinary farmer that are in danger of confiscation by the tanking organization in Chicago. There is far more "lump jaw" among the cattle of the West than any single owner has any realization of, because few, if any of them, have taken any pains to acquaint themselves with the actual condition. A very conservative estimate would be to say that one out of every five hundred head has this trouble to a more or less marked degree. I myself would say that there was at least one in every two hundred and fifty. Cattle men must, or should, be aware that the term "lump jaw" by no means means "lump jaw" in the minds and eyes of the Illinois confiscating brigands. To them almost any kind of lump, if no bigger than a marble, anywhere around the jaws, or between them, or along the neck, gives a sufficient warrant for the confiscation of the entire carcass and the supposed condemnation to the rendering tank. Whether all such animals really get there would seem to be a very doubtful question from testimony given at Peoria. The confiscation has been painfully evident to many a breeder by the depleted condition of his pocketbook. While to very large feeders the confiscation of two or three head of fine fat cattle in a large number sent to Chicago is not, or may not be, a very serious matter, it is undoubtedly so to the small feeder who takes in a car load or so, and it is these small feeders' losses which help to swell the aggregate loss, by this senseless confiscation, to a very large sum. But that is not all the loss. The fact that cattle with almost any kind of a lump, whether on the jaw or not, whether open and running or not, will be ruthlessly conficated in Chicago, has become pretty well known all over the West, and such cattle are now being used for home consumption at the cheaper prices of the grazing States, the owner thereby losing the profits of his feeding, the interest on the money invested as well as valuable time. It matters not how fine the condition of the animal may otherwise be, were it the crack steer of the yard, "to the tank" it must go by order of the great Illinois confiscatory. It is thus easy to be seen that the loss to the breeders and feeders is vastly more than is represented by the number of cattle confiscated at the stockyards at Chicago.

But that is not all by any means! There is still another side to this question of equal importance. For political reasons only, though successfully pulling the wool over the eyes of the majority of the farmers of the West, the present Secretary of Agriculture is making a great ado as to what he has done and is doing towards opening the markets of Europe to our live stock products. He does not, however, tell us of the real influences that have been at work, and still are, and will continue to work Europe in our favor. This year it is short crops and scarcity of food which are playing the chief rôle. The people are begging for bread and the governments have been obliged to roll away their obstructing stones somewhat from the doors. But a mightier power than the Secretary of Agriculture of the United States, the Republican party, the government of this or any other country has its hands on these barrier stones. This power is the wavelike and almost universal uprising of common, every-day humanity in every land, that great movement under many names, known as Socialism. It is *the people* who are opening the doors to our products. William II., of Germany, has shown that he hears and appreciates this demand better than any other European monarch or government, and it is to him more than to Secretary Rusk that the freer entrance of American meat products has been permitted in Germany. Socialism says "give us cheap bread and cheap meat." Socialism means mass-justice, rights and right to all, not communism or any such half-way theoretic affair. Socialism means inter-nationalism, not individualistic nationalism. It means the world and its products for humanity, not for Jew or Gentile, American or European. It means universal and free communion between all nations and all people, and the greatest possible distribution of their productions, so that each and all may equally share in the benefits of mutual production.

That "lump jaw" is neither a contagious nor infectious disease will be completely demonstrated in this paper, as well as that the flesh from such cattle is generally as fit for human consumption as any other animal product.

Lump jaw is neither a contagious nor infectious disease. It is necessary that we come to a clear understanding of what is meant by diseases being contagious or infectious. The discussion will naturally be considerably of a grind, and must necessarily be of both a scientific and practical character. It is absolutely essential, however, that the live stock men of this country, as well as the medical profession and lay public, should have a most definite and positive idea on this matter, and that so far as possible it should be settled once for all. This I shall do for every man of good, sound, practical, common sense. In fact, I dare make this assertion, that the practical, every day, intelligent and thinking citizen has clearer, more logical and more correct ideas of the meaning of the word "contagious" than the majority of the medical profession. Once, some forty years ago, before the birth of modern experimental medicine, especially the bacteriological craze, medical men also had some common sense, and knew the meaning of the word "contagious," but to-day it seems to have no definite meaning whatsoever; that is even more true of the great investigators than of the rank and file of the profession, though the latter have been pretty generally led astray through the erroneous teaching and influence of this modern school of experimental medicine, the workers in which " know the right, but still the wrong pursue." It is undeniably true that of all the so-called scientific professions, which have their appropriate and absolutely essential "termini technici," that the members of the medical have the least knowledge of their true and logical meaning of any. I will go so far as to assert that the average graduate and many of the so-called " professors " in our medical colleges, editors and writers in our medical journals do not use rightly, or even know the correct use of a vast number of the strictly technical words common in pathology I know full well the correctness of this assertion. How often have I heard that great master of pathology, Virchow, most terribly denounce this very ignorance while listening to his lectures for several years in Berlin, and more especially in many hours of private intercourse. It was from the master that I myself received not only the inspiration, but the instruction, which has made me, like him, somewhat of a dogmatist on this question. English and American medicine are both notoriously loose in this regard, and it is this very looseness of thought and expression which has given the Illinois Live Stock Commission its grounds for the utterly baseless assertion that "*Lump jaw is a dangerous contagious disease.*" The real fact is that every member of that commission, every veterinarian who certified on their side at the late trial at Peoria, not only knows that actinomycosis is not a contagious disease, but absolutely gave testimony in support of that fact. One of their chief experts, whom to avoid personally I will not call by name, when discussing this very question with me, said: "*I have put myself on record in that way* (meaning in the official report of the commission) and *must stand by it to be consistent.*" Which in common, every day language means, *he had knowingly lied in one place, and must keep on lying so as not to lose his job.* As hygienists, that is as members of any official body having "Sanitary Veterinary Police authority," the veterinarians of the Illinois Live Stock Commission know full well that lump jaw is not a "contagious disease" in any sense of the term. Practically they not only know it, but their every action in connection with lump-jawed cattle is positive evidence that they never did, and do not now consider it to be a contagious disease.

Now, let us see what we mean by a contagious disease. The classification of disease of certain types as either contagious or infectious is very old, and antedates by some centuries the birth of modern scientific medicine. It was a purely practical differentiation, based entirely upon daily observation and years of experience with the great pests which overran Europe in the centuries previous to the present, and upon the result of those observations and experiences comes this classification, which is entirely hygienic in its character, and expressed and still expresses the basis upon which all preventive measures in reference to contagious diseases should and must be put into execution. This hygienic practical division of diseases was not founded upon studies made in laboratories at a distance from diseased or better affected centres, and with very little regard for the daily, practical course of such disease among the people, or in stables and in fields as we say of animals, as is now too much the case (in fact, it would seem as if this side of the picture, and the experience of the ages, was almost neglected at present by so-called scientific investigators), but which was the only way diseases were studied, or could be, is those early days, hence the observations of the physicians of the sixteenth, seventeenth and eighteenth centuries on the real character of diseases, of their true nature, were often more exact and their conclusions more reliable than those of the men of to-day whose intellects seem to be clouded by the mere results of laboratory investigations, which in themselves do not necessarily teach a single thing about the chief character of a disease from a hygienic point of view.

The words contagious and infectious were originally, and in all intelligent hygienic bodies are still used to express the primary origin of given diseases. They indicate as plainly to-day as they once did the sources from which we may expect danger to the health of the community or our stock. The word contagious is from two latin words, one of which means "*with*," and the other "*to touch*," in other words, *to come in contact with* a given something.

What is that something?

*A living individual, let it be human or animal, in which a certain disease originates—exists.*

The old, as well as the modern hygienists said and say, that in order that a contagious disease shall not gain admission to the country, or transportation from an infected center over the country their efforts and observations must be entirely directed to individuals, or the possibility thereof, having such contagious diseases gaining entrance to the country. In other words, the word "contagious" is a genetic one; it points to the primary origin of the disease; it expresses the fact that to diseased, or in diseased individuals, we must look for the generation of a disease of this type *and all hygienic measures adjusted to isolating such individuals in such a manner that healthy ones cannot possibly come or get in contact with them.* That is, contagious diseases do not develop de novo, and so far as we have any historical record of their nature and course, have their primary origin in animal life only, be it in man or beast. Every stockman knows that! Every intelligent citizen knows it far better, it seems, than the medical profession of to-day, if one is to judge from the manner in which the word contagious is used in the writings and utterances of physicians. This Illinois Live Stock Commission knows it too, just as well as it knows that they speak falsely when they assert actinomycosis to be a "dangerous contagious disease."

A contagious disease then, is one which finds its only and primary origin in the previous disease of some individual of some susceptible species, and never, primarily, in any other way. In this manner such diseases have been kept in existence, and in this manner transmitted from generation to generation through the ages, as the "sins of the father have been visited on the children even to the tenth generation," aye to the thousandth. Giving attention to this fact only, to the diseased individual as the primary cause, the dangerous elements to be combatted, the hygienists of the past centuries have rendered our lives almost absolutely exempt from the dangers of such diseases as the black death and bubo pest which were once a terror to all Europe. We all know that we are in absolute safety against small-pox if there is not a diseased person in our section of the country, and none are permitted to come into it Every man knows, excuse the freedom, that if he does not go on a lark in his visitations to conventions, or on business in any large city, that he is absolutely safe against the greater pox and another female complaint, just as well as he knows that if the woman is healthy he is in no danger at all. We know that the avoidance of contact with persons having diseases of this fixed contagious type is sufficient to keep us free from such diseases.

Let us turn to animal diseases of this character for a few illustrations.

We all know that we have no dread of glanders among our horses if there is no horse afflicted with the disease in our section of the country. If there is, we kill that horse, and if the only one, after moderate cleansing and disinfection we feel safe once more. We know that we ourselves are in no danger whatever of glanders, no matter how closely we come in contact with healthy horses, while many a man has sacrificed his life either ignorantly or stupidly by fooling with "cures" for glandered horses. To-day we of the great prairie States once more sleep peacefully regarding any danger to our cattle from pleuro-pneumonia, but such was not the case when it existed in Chicago, and not only this State, but every western State, quarantined against Cook county, Ill., cattle. Why did we quarantine against the cattle from Cook county? Because contagious pleuro-pneumonia finds its only origin and only cause of spread in diseased cattle, because the disease is a contagious one. This very Illinois Live Stock Commission deserves the highest commendation of the entire live stock breeders of the country, for the energy with which it went to work and stamped out the lung plague in Illinois, even in spite of the lukewarm support of the Agricultural Department at Washington, as much as that department deserves the most determined and energetic condemnation for the way it nurses and supports that same plague around New York when a similar energy to that displayed by the Illinois Commission could free the country absolutely from the stigma of having such a dangerous contagious disease among our cattle. Once "stamped out" in this country, once sure that not a single case of the contagious lung plague existed in the country, we should all feel safe, but would equally well know that it could never come again *unless a diseased animal was imported from Europe*

In contagious diseases our chief attention is directed towards the diseased animal. It, or he, is the source of danger.

It is because of this that the rinderpest seldom visits western Europe at present, and has never been imported into this country. The animals do not live long enough to get here. In eastern Europe a constant watch of military precision is kept over the importation, or smuggling of cattle from Russia, the home of rinderpest. The Illinois Live Stock Commission pronounce lump jaw to be a "dangerous contagious disease." Is the lung trouble any more than that? At the late trial it was amusing to see the witnesses for the Commission hastily deny, or enter a protest if the lawyers for the plaintiff spoke of lump jaw as a "highly" contagious disease. As most contagious diseases that we know of are "highly" dangerous to life in a shorter or longer time, and "highly" dangerous to the well being of the community, it was rather singular to observe how "highly" sensitive those persons were on that point. Why has that Commission not put in force the laws and regulations it is empowered to do in reference to all contagious diseases in live stock, in its treatment of lump-jawed cattle?

The law certainly draws no line of distinction between "highly" and "dangerous" contagious diseases. It simply and plainly authorizes such Sanitary Commissions to act to the full extent of their authority in all cases of contagious diseases. What does it authorize them to do in such cases? To kill glandered horses, to kill, or dispose of all cattle afflicted with pleuro-pneumonia; to peremptorily stop all traffic in or with such animals, or all which have been in contact with them, as suspects, to quarantine against any State, or locality, in which such diseases exist according to their character and the common danger to the community; to burn, tear down, cleanse or disinfect all premises or vehicles where such animals have been; to put the necessary limitations upon the free movements of human beings who have been in contact with such animals, or where they are or have been. Briefly stated, such are the actions we have a right to expect, and for which they have been created by law of any Sanitary Board, be it for the protection of human or animal life.

Has this Illinois Live Stock Commission done any of these things, except ruthlessly slaughter the animals, in reference to lump jaw? Has it sent due notification to the governments of other States that they have a "dangerous contagious disease" in their cattle known as lump jaw, or actinomycosis, and such cattle will not be allowed to enter the territory of Illinois? Has it notified the commission houses in due and legal form to warn their patrons against shipping in cattle afflicted with this dangerous contagious disease? Has it isolated the balance of a car load when one or two lump-jawed steers are among the others, or taken one single step to protect the other cattle in the stock yards from contact with animals having this dangerous contagious disease? Has it cleansed and disinfected stock cars, or the pens in the stock yards? In fact, has it put in action one single preventive procedure that it actually would have done had a real contagious disease of any kind been among the cattle shipped into Chicago? Being such a "dangerous contagious disease" to both cattle and men as they claim, have they ever warned the poor unfortunates in the stock yards, condemned to work in such a pest house, against the terrible danger they were not only exposing themselves to, but nightly carrying home to their families? I have seen these men around among these cattle themselves; I have seen them cutting up and examining them, yet I never saw them exercise a particle of caution, or warn the men engaged in slaughtering them to do so; in fact, they all acted as unconcerned as if they were sitting down to a good meal; no one present seemed to have an idea that the cattle being killed were afflicted with a dangerous contagious disease, or dangerous to man or beast.

*It is not speaking too emphatically when I assert that every action or want of action of the Illinois Live Stock Commission absolutely and unequivocally gives evidence that they do not really believe one single word of their assertion that lump jaw is a contagious disease, as they manifest it in every act they do when combating diseases which really have that character.*

Furthermore, when on the stand, and under oath at Peoria, every one of their veterinarians, so-called experts, testified it was his belief as well as generally admitted by all who had any experience in the matter, *that the primary origin of actinomycosis, the original habitat of the fungus, was in some form of grain or grasses at present not positively known.* That one admission knocks their assertion that "lump jaw is a dangerous contagious disease" into smithers.

Did any mortal human, since history's beginning, ever hear of, has medical investigation in any land ever discovered, or is there any record of a contagious disease having its source of primary origin in a parasite effective vegetation of any kind?

I challenge the record on that point. Let us now turn to another side of this question for still more positive evidence that actinomycosis is not a contagious disease.

*Facultative Parasitic Diseases.*—The true and scientific, as well as practical hygienic reasons having been given for designating certain diseases as contagious, or obligatory—parasitic, because their germ cause only finds the conditions of nutrition suitable to to their own lives, in the chemical constituents of animal organisms, we now come to that other great class, the "facultative parasitic," or the infectious diseases of ancient writers. The term facultative-parasitic should carry its own meaning to every intelligent reader; it is that the germs causing this class of diseases are not of necessity bound on animal organisms, or their tissues or fluids, for their biological necessities (food); they are not obliged to be parasitic, that is, living on or at the expense of other organisms. They can live there, that is all! They have the faculty of adapting themselves to the nutrient conditions afforded by living organisms. Their natural habitat, however, is the earth, cesspools, earthy material, that is, in conditions offered by the surroundings of animal life. The diseases caused by such germs are known as Exogenous, generating from without the animal organism, or as I have termed them Extra (outside) Organismal in origin, which is the same thing. Here, then, we have the only essential difference between a contagious disease (Endogenous) and an "infectious" disease (Exogenous) in the sense of old writers and in the sense of hygienic or preventive medicine. The modern words correctly and logically express the true meaning, while the older ones do not, unless we know what we are writing or talking about.

Otherwise the two classes are very much alike in many points.

Nutrition plays as essential a rôle with regard to the germs of the facultative-parasitic or exogenous diseases, as with the obligatory-parasitic or endogenous, but with far more freedom of selection, or adaptation to surroundings. These facultative bacteria first of all have not that delicacy of constitution which requires that the temperature in which they live shall be nearly constant at one point or vary but a very few degrees from the same, that is, the temperature of the animal body which is from about 99° to 102° Fahrenheit. On the contrary, they will develop in any temperature, from a few degrees above freezing to the highest temperature of the animal body. Again, they are by no means so select in their choice of food; they develop in the natural earth, in filth, on and in dead bodies, as well as in the various artificial media, with considerable ease and certainty. In some things, however, they are very delicate in their selection of food, or the conditions under which they develop, but this is equally true of the obligatory germs. Most of them do not thrive in acid media food, nor do they in one having a very strong alkaline character. Some require oxygen to live, others do not, while still others will live with or without it; some will not grow at all in oxygen, while others require hydrogen gas to develop, and still others require carbonic acid gas, which is poisonous to animal life. It is thus to be seen that germs, like higher organisms, may be quite select in electing the conditions under which they live It is my opinion that the germs of the eruptive fevers (scarlet, measles, etc.) in man require carbonic acid to develop in, as they do not thrive well in the blood, but produce their chief lesions in the cutis, where the circulation is the poorest and the accumulation of carbonic acid the greatest. I think it is because investigators having the opportunity have not persistently tried to develop these germs in this gas that they have failed to cultivate them. As we ourselves only take such food as we want, and the digestive organs of our bodies can only handle that which is useful to us, so, like the Pharisee in the Bible, these germs "pass by" on the other side of those things which they do not need as food; they select what they want and let the rest alone. The trouble with us investigators is, that we cannot always select what the germs want, or so adapt the food we offer them, to their necessities, that they can select the useful and leave the balance alone; we shall improve as we continue to learn. We can change the character of germs by prolonged exposure to certain kinds of food. We can increase their so-called virulence, disease-producing power, or take it away altogether by changes in the conditions which we offer them to live in. Sometimes it is another degree of temperature, and again changes in the chemical constituency of the food we offer them. In one case at least a natural accident has changed the character of a germ so as to make it invaluable to the preservation of human life. I allude to the germ of small-pox. When considered as the germ of that disease we all dread it for its extreme virulence, giving to the disease its specific contagious character; but when we think of vaccina, cow-pox, it is not with a feeling of dread, but of grateful thankfulness to the genius of the immortal Jenner. We have no dread of vaccinated children. The germ of the disease in man and cattle is the same, but through the accidental transmission (perhaps for centuries before Jenner made his observations, when small-pox itself was one of the terrors of the world), of the disease from cow to cow (milk cows generally have slight abrasions of the skin of their teats) for generations by the milkers, a different medium chemically (food) was constantly given to the germ, in which it thrived, but so changed its character as to lose its entire virulencet it did not produce a contagious disease in cattle or man, and does not to-day, *though it can be transmitted* to either, and indefinitely further in healthy individuals of either species. This fact is especially called to the attention of those imbecilic, illogical or ignorant medical men or investigators who assert *transmission to be identical with contagiousness*, I speak in the genetic sense only. Something on the other hand was retained in this bovination of small-pox. That invaluable something is the preventive power (without virulence) of vaccina. Nature has done, through the accidental and unreflecting assistance of man in this one disease, that which is at present beyond the power of man to do with the known germ of any other non-recurrent disease of man or beast. As said above, we can do what we please in regulating the virulence of known germs by changes in the conditions we offer them to live in, but while the germs of non-recurrent diseases must necessarily also have a preventive power (or second attacks would be the rule instead of the exception) we know of no means of so cultivating such germs artificially, as to preserve this chemical protective product without the virulence. Could I only do that, the problem of an absolute and constantly unvarying preventive virus against swine plague would be solved. I do not despair. It is objected by the Bureau of Animal Industry that I publish almost entirely for the farmers and in public prints. That's queer! for whom do they issue their reports? This work is paid for by and being done for the people, not for the self-congratulatory amusement of a few investigators. It is certainly not being done for the edification or education of the government dilitants, though they are slowly beginning to learn its value and to profit by it. If Nebraska continues to support it they may eventually learn something in Washington.

It will be remembered that we are discussing those germs which are not necessarily the cause of infectious diseases, but which have the faculty of becoming such. These are exogenous germs; that is, such as primarily live and find their natural home outside of any animal organism. In reality they are far more important than the obligatory parasites, or the germs which thrive only in the animal body.

The study of their lives is also far more interesting. While the obligatory parasitic diseases have been lessened in number by our hygienic endeavors, and are being brought under more direct control, the facultative parasitic are in danger of being increased. The cause of this is to be sought in nutrition. It is one of the penalties of civilization, of ignorance and carelessness of the aggregation of animal life in one place and grouping densely; of the saturation of the soil with animal excretions, urine and fæces. This group may be called *the penalties of civilization.* Another group may be termed *the natural facultative parasitic diseases*, because they are not directly produced by the influence of any act of man, though they may be transported from place to place by him as any other seed can be. They are spread like our weeds. Here again, civilization pays its penalty. Let us tabulate a few of these two groups and then consider them apart.

| NATURAL FACULTATIVE PARASITIC. | ARTIFICIAL FACULTATIVE DISEASES. |
| --- | --- |
| Yellow fever. | Typhoid fever. |
| Texas fever. | Diptheria. |
| Fever and ague. | Rouget of swine (in Europe) |
| Swamp malarial fevers. | The horse distempers. |
| Anthrax. | Swill and filth diseases in swine. |
| Black leg. | Cerebro-spinal meningitis. |
| Mad itch. | An unnamed kennel disease in dogs. |
| ' Wild-Seuche" in swine cattle and deer. | Hen cholera. |
| | Swine plague. |

What then is this difference?

It is plainly evident if we give our attention to both these groups of facultative parasitic diseases, as they occur under natural conditions, "in the field" as we say when speaking of the study of animal diseases. It cannot be discovered by any laboratory investigation, however, the misfortune of which has been to so magnify their importance that the real study of diseases under the conditions in which they daily occur has been pushed into the background, and too frequently neglected altogether.

In facultative parasitic diseases of the "natural type," it is evident (illustrated by yellow fever, Texas fever, anthrax, etc.), that the saturation of the soil with animal excreta is not necessary in order that the bacteria causing them shall be virulent to man or beast. These germs are naturally so. They find the conditions to such virulence in the nutrition offered by the virgin soil itself, under certain local chemical and climatic conditions. They find equally favorable nutrition in the organisms of man or susceptible species of animal life.

The other group is entirely different. They inhabit certain forms of virgin soil, and find their natural home also under certain local and climatic conditions. But under such circumstances they are as harmless as a cooing dove. Man may take them into his system (the same of animals), and they pass out again doing no harm; but when man has settled on such soil, and it has become saturated with the chemical products of human as well as animal excretions and filth, these germs change their character ; they become virulent, in other words, they produce a substance themselves, which experience shows is toxic or disease producing in certain forms of animal life and not in others. Why is this ? Attention has already been called to the fact, that like every other living organism, germs are also selective in reference to their food ; they pass by that which they do not want. Regarding this virulent, toxic, or disease producing property, it has also been noticed that we ourselves can influence it very easily in any direction by changes of food in the laboratory. It is not too much to assume that the same thing occurs in nature, though it may have taken centuries to bring it about, and has not come about until after years of saturation of the soil with the chemical elements of the animal body passed off in the excretions. A careful study of the diseases afflicting man in his most natural, simple and isolated condition, will, I think, most successfully demonstrate the correctness of these conclusions. In this class of diseases, as well as the natural facultative, and also endogenous diseases, we also find this elective, nutritive attribute displayed, in that certain germs gain entrance into and grow and produce disease in certain species of animals, while others that are fully as much exposed to their action are not in any way injured. In swine-plague in a herd of two or three hundred hogs, the animals eating the same fodder, polluted with their excrement, given an equal number of cattle, it is absolutely impossible that the cattle should not also take the germs into their intestines ; so of horses in the same yard, and I have seen hens and ducks eating their fill of dead hogs many a time, and yet not one of these other animals ever sickened in hundreds, yes, thousands of such experiences in this western country.

The most striking example that has come within my personal observation of the influence of changes in nutrition under natural conditions is given by the practical result of the disease of corn to which I have given the name of "Corn stalk disease," and in which I successfully demonstrated the germ during my previous term of service in Nebraska. So far as practical experience and observation extends, the germ of this disease of corn (it has been described by Burrill, of Champaign, Ill.) though manifesting itself in the green and growing corn, is absolutely harmless to cattle or other susceptible stock, so long as the corn is green. It is only when the corn is dead, towards the latter part of October or in November, that the germ in the corn becomes dangerous to cattle. Basing my hypothesis upon this annually occurring practical observation, I

assumed that during the slow process of the death of the leaves (the germ in virulent form has as yet only been demonstrated to exist in the leaves and husk of the cob the chlorophyl undergoes certain chemical changes, thus altering the nutrient conditions offered to the germs and supplying them food out of which they secrete some toxic or disease-producing stuff, which property is also retained by them when they gain entrance to the bovine organism.

It seemed very essential to endeavor to prove this to be so by actual experimentation. Thanks to the publication of Prof. Burrill, we were enabled to recognize the disease in the growing corn, a plot of the same having been discovered in the past summer by Mr. Duncanson, assistant to Prof. Ingersoll, Agriculturist of the University of Nebraska, and with the co-operation of Prof. Bessey, of the same institution, I was enabled to get what proved to be the desired material, a full description of which, with most exact illustrations, will eventually appear from the hands of that accomplished botanist.

The first corn brought to me was green, though somewhat discolored in spots ; but it is not my province to describe the lesions presented. From the lesions I isolated a germ which, so far as could be judged, was the same described by Burrill as well as that previously obtained by me in the organs of cattle that had died after being turned into stalk fields. The inoculation of rabbits with pure cultures of this germ at this time (August, 1891,) as well as feeding others with the diseased leaves, produced no effect whatever. Towards the last of September another batch of stalks from the same field was brought to me, more discolored and wilted than the previous ones, but still sappy and greenish. The same germ was again isolated and similar inoculations and feeding experiments made, with the result that the rabbits were slightly ill and off their feed for a day or so, but nothing serious. On December 10th, Mr. Duncanson again brought me stalks from the same field, this time both stalk and leaves being dry and dead. No attempt was made to isolate the germ from the dry leaves, but they were stripped and fed to rabbits, plenty of water being given them. Two rabbits died on the 12th of December, their blood being replete with the previously mentioned germ, as shown by comparative cultures in and on different media, though I consider the source of the germ and the well-known conditions under which it was obtained, and the experimental results, the only reliable data upon which to establish identity in such cases.

These results, I think, are not only very instructive, but absolutely conclusive of the correctness of my previous hypothesis as to the changes in the chlorophyl causing the change in character of the germ, through its changed food, thus making it disease producing in cattle.

To the feeder they say in no uncertain language, raise fodder for your stock, cut it green and do not turn it into stalk fields until you can learn how to distinguish the desired corn and cut it out. This is another strong point for the silo ! This corn fodder disease is transmissible from animal to animal by inoculation. I should like to see the fanatic who would dare affirm that it should therefore be classed as a contagious disease.

It has almost been impossible to satisfactorily produce typhoid fever in any of our domestic animals, and as a natural disease, it does not occur in them. This immunity of one species to the germ causing most malignant disease in another, can be daily demonstrated by actual experimentation. Even a more singular thing is that germs can be isolated and cultivated from the intestines of animals which while there do them no harm, but which if injected into the blood of an animal of the same species it will kill. Then there are a large number of germs in the mouth or intestines of animals that are experimentally fatal if inoculated into one or the other species of small animals used in laboratories, and yet are harmless where originally found.

These artificial facultative parasites only cause specific disease (become parasitic) in that species of animal life, the chemistry of which organisms is the same as that of the excreted chemicals in the soil that have changed these same germs from non-virulent harmless organisms to most virulent facultative parasites. This saturation of the soil with animal (human) excretions is the real cause of typhoid fever, diphtheria, swine-plague and the diseases of this group; hence, they may rightly be termed the penalties of civilization. It is the same conditions which cause the fatal stable diseases in horses in our city stables. This is why cities like Chicago are fast becoming unfit to live in, and why we are learning to give so much attention to sewage and drainage and the care of all excreted materials. Plenty of sun and the most perfect and deep-seated drainage possible with the most complete removal of all the excreted materials of animal life, must be the aim of our hygienic endeavors. Swine-plague was never imported into this country. Its germ is a natural inhabitant of our moist heavy soils, but not of our sandy, gravelly or shallow soils. When we began to raise hogs in great numbers and for a series of years in these sections of the country the swine-plague first appeared and has been growing worse ever since, in such sections. In sections having a dry, sandy or gravelly soil it does not primarily originate, the germ is not there, or if there, the nature of the soil is much that it does not hold these chemical elements in sufficient degree of saturation to change the character of the germ. Even if the malignant germ is carried to into such sections by infected hogs, the disease does not become permanently planted there.

To sum up : *A "contagious" obligatory parasitic or endogenous disease, is one that finds its only source of primary origin in a diseased individual of a given species of animal life, while an "infectious" disease (in the ancient sense and hygienic meaning of to-day) is a facultative parasitic or exogenous disease, or one which as invariably finds its primary origin in conditions outside the animal organism.*

From this genetic, primarily hygienic point of view, every other characteristic of these diseases is of no importance whatever.

*The Word Contagious a Humbug.*—The resemblances, or similitudes between endogenous and exogenous diseases.

We come now to the discussion of the contagious in an even more exact manner—in order to show that unless used in the pathogenetic, or primary disease producing sense, already so fully explained, that the word is a miserable one, and in any other sense has no scientific or special meaning whatever. So misused has it been, and still is, its application that the best thing would be to draw the word "contagious" from medical literature altogether. Taking the roots of the word itself from "*con*"—with, "*tangere*" —to touch, or to come in touch or contact with a given something, it is easily to be seen that the word has no particular or limited application whatever, and this is the reason that its meaning has become so indefinite in modern medicine that a person must have a clear and positive idea of the whole subject another is talking or writing about, before he can form any definite conclusion as to what such a person means when he uses this abomination to every educated and logical student of the phenomena of disease. Let us see how absurdly, yet how justly, according to its root derivation, this word "contagious" can be used. You go into blacksmith shop and carelessly pick up a horshoe from the floor, that has cooled off enough to look black, and it burns your hands severely and an intense inflammation follows. It is absolutely correct for you to say that that piece of iron was a "contagion," and the resulting inflammation a local contagious disease, for did you not come in touch with it, and did it not produce disease? You go out of a night and drink too much whisky, and derive an acute inflammation of the stomach for your dissipation. The whisky is a contagion and the inflammation contagious, for your stomach comes in contact with too much alcohol. In both cases you acquire an inflammation through coming in contact with a given something. Now, an ordinary layman would never for a moment think of speaking of any disease acquired in that way as contagious, yet it is with just such absurdity that the word is used by otherwise well-educated and unquestionably competent physicians.

It will be remembered that it is on the ground of the possible transmissibility of actinomycosis, from an animal afflicted with lump jaw, by the act of inoculation in the hands of an experimenter, that the Illinois Live Stock Commission have pronounced lump jaw to be a dangerous contagious disease, or to use their own words:

"*It was clearly demonstrated that the fungus—actinomycoses—could be transmitted from one animal to another by inoculation, which fact establishes it as a contagious disease.*"

"This fact was never scientifically and surely demonstrated until 1891."

These modern methods of experimental transmission in small animals have come in vogue because investigation has gradually led to the discovery of the actual causal moment in many diseases, and found them valuable (to a degree) in studying the development of such moments—germs—and not that such transmissions had a particle of necessary relation to the primary origin of a disease, or its actual course in that species, (or those) in which, under natural conditions, the germ is the cause of a specific disease.

Long before even the great John Hunter performed inoculations between persons—and from a primary lesion (chancre) to a healthy portion of the same person in syphilis, it was as evident as then that the disease was contagious, and found its origin in man only, and passed in some way from man to man. The combined failure, by all investigators, to transmit syphilis to other animals save man, and produce syphilis, in no way influences the well-known fact that syphilis is an endogenous (contagious) disease. Do we need any laboratory, or experimental proof, in any other animal, or even any experimental transmission in the horse, to strengthen the fact that glanders primarily originates in the solipeds

—horses and asses? It is self-evident that we do not! Does the fact that glanders is not transmissible to cattle alter the natural condition at all, so far as the origin of the disease in solipeds only is concerned, and is its contagiousness any more strengthened in that species because it can be transmitted to guinea pigs, rabbits, man, dogs and other animals? Is pleuro-pneumonia of cattle any the less a contagious, endogenous disease in the bovine species, because it has never been transmitted to any other form of animal life, either accidentally or experimentally, so far as I know, though if I remember correctly, some form of pleuro-pneumonia has been said to occur in goats, but there is no proof that was the specific contagious disease of cattle?

Even in the inoculation of cattle against lung plague the specific lesions in the lungs are not produced. According to such authorities (?) as have been quoted, there is no proof of that disease being contagious in cattle, for the "crucial test" is wanting. Because some inflammatory product can be carried into the "conjunctival sac" of the eye socket, as Burdon-Sanderson asserts, is not a particle of proof that there is anything "contagious" in a simple inflammation, even though we can isolate one or the other of the pus-cocci from the inflammatory products produced, and inject the culture under the skin of a susceptible small animal and kill it, still that act of transmission may be, and generally is, absolutely without value in throwing any light on the primary origin of the coccus, or the nature of the inflammation in the original subject.

Every practical act of any hygienic body of competent physicians completely contradicts this laboratory-transmission nonsense. We look first at the source from which the danger originally came, and then at the chances for transmission from the diseased individual to healthy ones, but the "transmissibility" bears no necessary relation to the primary origin, and no disease on earth can be classed on such a foundation, nor differentiated from another.

There is not an observer or authority who must not and will not admit *that all contagious diseases are infectious; but there is equally well not one who would dare affirm that all infectious diseases are contagious.*

What constitutes the difference? On what characteristic of these diseases does this universally admitted differentation bear, and still is based? Had the fact of transmissibility either in the species in which a natural susceptibility exists, or the experimentally demonstrated possibility of transmission to rabbits, guinea-pigs, or what not, an iota of influence in determining this universally recognized differentiation of infectious diseases into two classes? Emphatically, no. Again I ask, what has caused and still causes such distinguishing between them?

Every one knows that this division is based on the primary origin—original source of danger—of the two classes, and not upon secondary possibilities. *Not to the diseased individual present, but to the source from which, in the first place, that individual became infected, and which has been, so far as is known, the only source from which or in which such a disease finds its primary genesis.*

*The folly of the use of the word "contagious" is illustrated by many endogenous and exogenous diseases having numerous common characteristics.*

"Contagious" logically means to come in touch with some infecting medium, nothing more. Contagiousness and transmissibility are one and the same thing. It has no necessary bearing on the origin of the agent transmitted, however, and as the word "contagious" has had an old and well-established meaning, which ignorance has partially robbed it of, it should be dropped altogether, as in its present use it is only misleading and false. This is best demonstrated by considering it as identical with transmissibility.

An example: A horse is turned out in a certain pasture in which is some low, moist land; the grass is dry and hard, except in this moist place; the animal seeks out this spot and grazes there, and in two or three days dies of anthrax; a veterinarian is called in to make an autopsy; his hands have some scratches on them, and he acquires malignant pustule, as anthrax is sometimes called in man. What has happened? The veterinarian has been in contact ("contagious") with a diseased horse; having had wounded hands, this contact has led to the transmission of bacillus anthracis (the germ) from the dead horse to the man. In the modern sense, then, anthrax is a contagious disease, because it is transmissible.

Our veterinarian recovers, and again is called in to examine a horse suspected of glanders. Again, through some accident, he has wounded hands, and in feeling around the nostrils of the horse, gets some of the disease—secretion—in the wound. By this accident of contact and an opening in the cutis, he has come in contact with the bacillus of glanders, which has thus been transmitted to him. He dies this time, though that matters not in the question. Here again we have contact—contagious—and transmission.

Who will dare tell me that anthrax and glanders are diseases of the same character because of this identity in the characteristics of transmission and infection? How then, where then, shall we look for a logical point of differentiation?

To the seat of primary origin of the respective germs in each case. The horse that had anthrax found the bacillus in the soil, hence it is an exogenetic disease, bacillus, but has the faculty, however, of living in some form of animal life. The fact that from that horse anthrax could be transmitted to healthy horses or rabbits, and cause the disease in each one, is of little or no value, however. We can stop the danger by burning the first horse up and keeping others out of that portion of the pasture or fencing it tightly up, or in time by good drainage. The glandered horse, however, acquired the disease from another horse, that is all. We must trace the transmission back from horse to horse until we find the first one, and that being the last glandered horse in the country, we can make an end of the disease, if no other gets in. Who cannot see the vast difference, and that the word "contagious" in the modern usage is an insult to common sense?

This is why *all contagious diseases are infectious*, *though all infectious diseases are not contagious.*

What is an infectious disease? As it is now admitted, even by the Illinois Live Stock Commission, that actinomycosis finds its primary origin in vegetable life, and as it has been demonstrated that any disease having that character or origin cannot possibly be "contagious" in the only sense in which that word can be logically used in medicine, it is evident on that basis that the Illinois Commission has not an iota of authority to condemn a single steer, no matter how many lumps he may have on his jaws.

The question now before us is: *Actinomycosis not being a contagious disease*, *is there any evidence that it is an infectious one?*

It will be remembered that it is generally admitted that, while all contagious diseases are infectious, all infectious diseases are by no means contagious.

This very admittance, which is absolutely recognized by every physician—that is, that there is a difference—completely knocks the ground from under the assertion that actinomycosis is a contagious disease. *Now it is submitted*—evidence enough has been and will be also submitted—*that transmission, either natural, accidental or experimental, gives no logical ground upon which to differentiate infectious diseases, and that it is not now and never has been the reason which has influenced medical observers from the earliest dates to reeognize the fact that "while all contagious diseases are infectious, all infectious diseases are not contagious."*

We have now to consider what is the generally admitted and specific characteristic of an infectious disease.

It is this, *that some living organism—a germ—gains entrance to an animal organism, and lives and develops therein, during which time it produces a specific something (poison), which, being taken up by the blood, causes a general pollution of the organism, that is, a general disease, which is manifested by certain lesions common to all infectious diseases.*

The phenomena of this class are: First, fever, or a rise in temperature; second, certain changes in the great glandular organs, liver and kidneys and muscles, and musculature of the heart. These are the common lesions in which one infectious disease varies only in degree from another. On the other hand, each infectious disease has something specific to it, some organ or tissue shows itself to be peculiarly suited to the action of the germs; thus the eruption in scarlet fever and measles, the specific lesions of syphilis, the diseases of lungs in contagious pleuro-pneumonia, the ulcerations and other products in glanders, the lesions on the feet and in the mouth in the contagious foot and mouth disease. In actino we certainly have the local disturbances in the jaw, the tissues around it, and other parts of the body. The question is, do we have the general disturbances common to all infectious diseases? Do we necessarily have fever in actinomycosis? The second is, can actinomycosis be permanently cured by a surgical operation? for if so there can be no general disease. In other words, if the lump is removed, does the disease go on and display itself by other lesions over the body?

At the State farm we have a cow that has had actinomycosis for over five years, and been among the other cattle all the time, and yet there is no other case among them. We will only consider her as to that universally common phenomenon in all infectious diseases, *fever*, only.

For this purpose her temperature has been taken with that of two others in the herd every day for two weeks. It should be mentioned that a more extensive case of lump jaw has seldom been seen by any one.

| | SHORTHORN COW CALVED JAN. 18, 1885. | | SHORTHORN HEIFER CALVED JAN. 10, 1889. | | LUMP JAW COW BORN JAN. 7, 1891. | |
|---|---|---|---|---|---|---|
| | A. M. | P. M. | A. M. | P. M. | A. M. | P. M. |
| Dec. 1, | 38.80 | 39.20 | 39.50 | 39.30 | 39.30 | 39.10 |
| " 2, | —— | 39.10 | —— | 39.10 | —— | 39.10 |
| " 3, | 38.40 | 38.40 | 39.50 | 39.40 | 39 10 | 39 30 |
| " 4, | 39.10 | 38.65 | 39 80 | 39 30 | 39.20 | 39 00 |
| " 5, | 39.30 | 38.80 | 39 70 | 39.80 | 39.20 | 39.10 |
| " 6, | 38.70 | 39 00 | 39.70 | 39.60 | 39.20 | 39.15 |
| " 7, | 38.60 | 38.70 | 39.90 | 39.50 | 38.90 | 38.70 |
| " 8, | 38 90 | 38.80 | 39.90 | 39 50 | 39.10 | 39.20 |
| " 9, | 38.70 | 39 00 | 39.50 | 40.50 | 39.00 | 39.20 |
| " 10, | 38.90 | 38.90 | 39.70 | 40.00 | 39.20 | 39.80 |
| " 11, | 38.90 | 39.00 | 39.60 | 39.80 | 30.05 | 39 10 |
| " 12, | 38 90 | 40.80 | 39 90 | 40.10 | 38.90 | 39.30 |
| " 13, | 39 10 | 39.00 | 39.90 | 39.95 | 39.00 | 39.10 |
| " 14, | 39.00 | —— | 40.00 | —— | 39.20 | —— |

The above given measurements were taken, as can be seen, with a centigrade thermometer. The normal mean temperature for cattle being from 38 to 39.50, or from 101 to 102½° Fahrenheit. It is at once to be seen that the temperature of the lump-jawed cow was not any higher than that of the two looked upon as absolutely healthy, and not above the normal limits, while that of the heifer, as is usually the case, was the highest of the three. Here, then, we have conclusive evidence that nothing existed in the lump-jawed cow of a general infectious nature ; that actinomycosis, does not, itself, produce any specific chemical irritant, and hence, cannot be a specific infectious disease.

Again, it is now generally admitted that the actinomyces, when unpolluted, do not produce pus, so this bars out another condition of infection. When pus is present the pus germs are mixed with the actinomyces. It may now be said that actinomycosis is not an acute infectious malady, but rather a chronic infectious (contagious) disease. Let us, then. consider it from that point of view. Syphilis, while often an acute contagious disease, is also a very chronic one. In either case, however, *it is an infectious disease*, as are all contagious —endogenous—diseases. Some years ago it was asserted and most desperately hoped to be true, that if the primary lesion, chancre, was surgically cut out when it first appeared, that generalization—infection—would thereby be prevented. This idea has been completely exploded and the fond hope blown to the winds. The general infection invariably follows, though complete healing may rapidly result where the prime chancre was cut out, and nothing again appear at that point ; but the general infection as invariably follows, as said before. How is it with actinomycosis in this regard? Unfortunately, we have no exact and reliable data regarding the operation in cattle, but it is, perhaps, all the more fortunate that human medicine is replete in reports of so many cases of lump jaw, successfully operated upon, that it can be stated, as a law, *that wherever the actinomycotic lesion is so situated that it can be readily got at with a knife and completely removed, that a permanent cure will result.* On this point I have not yet had time to collect all the cases reported in the literature of our library, but a sufficient number have come to hand to answer every practical purpose, and that is all we need at present.

In the *London Lancet*, 1888, Vol. II., p. 176, two cases of lump jaw, one in a man and one in a woman, are given as successfully operated upon by Barez in Germany. In same journal, Vol. II., 1891, p. 1,115, "three highly interesting cases" are reported, and the editor says, "the chief lessons derived from them are:

"1st. The readiness with which the fungus may be detected in the discharges from the lesions.

"2d. The tendency of the growth is to localize and not disseminate.

"3d. The suggestion, almost amounting to a certainty, of the cause of the disease originating in cereals—vegetation."

All these recovered after operation.

Bostroem gives a number of cured cases: One of left upper jaw, five of lower jaw, and one of pharynx and adjoining tissues.

Senn, of Milwaukee, gives a cases of actinomycosis of the upper jaw in which complete recovery followed.

W. Fischer's case of actino of the tongue, was completely cured.

Baracz gives nine cases, mostly of the jaw, which were permanently cured where a radical operation could be performed.

Lunow says that of one hundred and fifty recorded cases, seventy-five have been cured, and gives four more cured cases, and Matta-kowski another. Israel reports three, Roser two, and Kepeller fifteen more, all permanently cured. Bertha, a case of intestinal actino cured. Helferich, two more in same locality, also cured; Leser, three cases of skin actino, also cured; and Koettnitz, three more cases of lump jaw in man permanently cured. Ponfick, in

1882, records sixteen cases, eight of which were cured. *Fifty per cent. cured.* The above cases are certainly numerous enough to show that actinomycosis cannot be classed as an infectious disease, in any sense of the word, *for the annals of medicine in all countries and all ages have no record of any infectious disease of any acute kind, or chronic, which can be cured by an operation in the above manner.*

Another very interesting and instructive point can only be seen by a careful study of the statistics. It is this : That very few cases of actinomycosis have been found in butchers or meat dealers in comparison to the vast number in agriculturists or farm laborers, or those having to do with grains or grasses ; in fact, the greater number have been in this class of people.

On the point of actinomycosis being an infectious disease, Bostroem says: "*I can only consider the changes produced by the actinomyces (the fungi) in the organism to be of a simple inflammatory character, and the products as only those of simple inflammation. The tissues produced by the action of the fungus I hold to be of a transitory character, which opinion is supported by their complete disappearance and healing, which often follows rupture of the abscess, as well as other reasons which contradict the idea that these products should be classed with the genuine tumors. I must protest against the classification of actinomycosis with the infectious neoplasms.*

What, then, is the nature of actinomycosis?

*Actinomycosis is an invasive disease, that is, a parasitic disease, in this case a vegetable parasite, which, on an opening being present, either natural or accidental, can invade the tissue and develop there because the nutrition is favorable, but which, of and by itself, never produces a chemical irritant which, by absorption or otherwise, gains access to the blood and produces a general constitutional disturbance, or infection.*

(*To be continued.*)

